# Closing Editorial—Special Issue on Veterinary Vaccines and Host Immune Responses

**DOI:** 10.3390/vaccines14010099

**Published:** 2026-01-20

**Authors:** Ayumi Matsuyama-Kato, Mohamed Faizal Abdul-Careem

**Affiliations:** 1 Department of Pathobiology, The Ontario Veterinary College, The University of Guelph, Guelph, ON N1G 2W1, Canada; 2 Faculty of Veterinary Medicine, University of Calgary, Calgary, AB T2N 1N4, Canada

As this Special Issue concludes, we are delighted to highlight the diversity, depth, and translational potential of the assembled contributions. These studies span multiple species (cattle, swine, poultry, and horses) and encompass a wide array of pathogens, viruses, protozoa, bacteria, and ectoparasites and vaccination strategies, including live-attenuated, recombinant, nanoparticle-based, inactivated/killed, protein subunit, vector-based, and commensal/microbiome-guided approaches. Together, the articles in this Special Issue present the state-of-the-art in veterinary immunology, zoonotic disease prevention, and One-Health vaccine research.

Key Contributions

Vaccination is key to controlling diseases in livestock animals and immune responses conferred by vaccines are critical to elucidate the protective mechanisms of vaccines. Santamaria et al. evaluated local inflammatory responses and systemic antibody production based on the intradermal injection of autogenous *Salmonella*-killed vaccines into growing feather pulps [1]. The research highlights killed vaccines that are able to elicit strong immune responses when properly formulated and delivered.

When vaccines are developed, the assessment of the welfare and performance of the vaccinated animals is essential. Silva et al. focused on the evaluation of commercial vaccines for nursery pigs against porcine circovirus type 2, *Mycoplasma hyopneumoniae*, and *Lawsonia intracellularis* and highlighted the importance of considering animal welfare and production outcomes [2]. The results indicated that although health and performance outcomes were comparable among all the vaccination protocols compared in this study, welfare and growth performance differed among the protocols, which suggests a suitable protocol during the nursery phase.

Tirosh-Levy et al. evaluated the impact of vaccination against neosporosis across multiple pregnancies. The study concluded that vaccination alone has a limitation to control the disease as neosporosis is influenced by environmental and infectious factors [3].

Natividade et al. demonstrated the protective immunogenicity of protein-based antigens derived from salivary and intestinal tissues against *Amblyomma sculptum*, the main vector of *Rickettsia rickettsii* [4]. This study proposed an effective strategy for vector control that can reduce both animal and human disease risk.

Wagner et al. examined vaccine-booster efficiency in equine mucosal immunity [5]. The study showed that intramuscular vaccination against equine herpesvirus types 1/4 in previously exposed horses boosted mucosal antibody responses, which emphasizes the importance of mucosal immunity in respiratory disease protection.

Calvo-Pinilla et al. proposed the heterologous prime boost vaccination strategy against Crimean–Congo hemorrhagic fever virus (CCHFV) [6]. Vaccination with protein nanoparticles and boosting with a modified vaccinia Ankara viral vector encoding the nucleoprotein of CCHFV demonstrated how modern vaccine platforms can elicit robust antibody-mediated and cell-mediated immunity and provided a framework for high-risk zoonotic pathogens.

Ramirez-Medina et al. demonstrated that the live-attenuated recombinant FlagT4G vaccine candidate showed genetic stability after multiple passages in pigs and induced protection at low doses [7]. The results emphasized its potential for field deployment and contribution to the control of a major swine pathogen.

Highly pathogenic avian influenza (HPAI), particularly the currently dominant clade 2.3.4.4b, continues to pose a significant and ongoing risk to global poultry production and public health. Abir et al. demonstrated that Nodavirus-Like Particles expressing the genetically conserved domain, M2e, have the potential to enhance immunogenicity as an avian influenza vaccine [8]. Kovács et al. summarized global vaccine trials and emphasized the need for the long-term observation of vaccine protective efficacy and the integration of vaccination with biosecurity measures to mitigate zoonotic risks [9]. Sang et al. summarized recent advances in next-generation vaccine design, microbiome–immune interactions, commensal vector-based platforms, and AI-driven vaccine development [10]. This review article underscores the critical demand for a One-Health approach to control the risk of pandemic emergence. As influenza viruses mutate frequently, the development of a universal influenza vaccine has become a major goal in influenza vaccinology.

Concluding Remarks

This Special Issue represents comprehensive research of current veterinary and zoonotic vaccinology, from molecular and experimental immunology to translational evaluation, welfare assessment, and foresighted review analyses. Together, these works chart a path toward safer, effective, and sustainable vaccines and emphasize welfare considerations, One-Health integration, and innovative platform technologies.

We extend our deepest gratitude to all authors, reviewers, and Editorial staff for their dedication and scientific rigor. Their collective efforts have produced a Special Issue that is both scientifically robust and globally relevant.
